# Health services research doctoral core competencies

**DOI:** 10.1186/1472-6963-9-107

**Published:** 2009-06-25

**Authors:** Christopher B Forrest, Diane P Martin, Erin Holve, Anne Millman

**Affiliations:** 1 General Pediatrics, University of Pennsylvania School of Medicine, Philadelphia, PA, USA; 2 Health Services, University of Washington School of Public Health, Seattle, WA, USA; 3 AcademyHealth, Washington, DC, USA; 4 Johns Hopkins School of Medicine, John Hopkins University, Baltimore, MD, USA

## Abstract

This manuscript presents an initial description of doctoral level core competencies for health services research (HSR). The competencies were developed by a review of the literature, text analysis of institutional accreditation self-studies submitted to the Council on Education for Public Health, and a consensus conference of HSR educators from US educational institutions. The competencies are described in broad terms which reflect the unique expertise, interests, and preferred learning methods of academic HSR programs. This initial set of core competencies is published to generate further dialogue within and outside of the US about the most important learning objectives and methods for HSR training and to clarify the unique skills of HSR training program graduates.

## Background

Health services research (HSR) is a scientific field that examines the structures, functions, policies, and outcomes of health services delivered to individuals and populations[1]. Its purview is global and involves investigation into all service sectors that affect health, not just the medical care system. Given the field's 40 year history, it is now appropriate to propose a common set of educational competencies. Elucidating the knowledge base and skills needed to be a successful health services researcher will aid in defining the field's professional identity.

### Need for Core Competencies

In the 1995 Institute of Medicine report, Health Services Research: Training and Workforce Issues [2], four elements of HSR training were emphasized: its multi-disciplinary approach; inclusion of basic and applied research; use of conceptual and theoretical relationships within and between health systems; and, research done on both populations and individuals. Although the report did not specify the core skills or knowledge sets that are common to HSR professionals, it did suggest that "a single educational path is neither practical nor desirable" and HSR programs should offer a range of training opportunities.

HSR incorporates a wide range of disciplines, notably biomedicine, economics, epidemiology, informatics, management sciences, political science, psychology, sociology, and statistics. Application of these disciplines to problems confronting health systems requires a workforce with a diverse skill set. The field has specific journals, professional societies, employers, sponsors, and training programs; however, the competencies common to all doctoral-trained HSR professionals have not been well-defined.

Graduates of HSR training programs pursue a wide variety of careers in academia, research, healthcare delivery, and policy analysis. Despite the success of these graduates, they complete their respective training programs unsure what it means to be a health services researcher, how they are distinguished from other professionals, and what professional roles their advanced training has prepared them to undertake. Our inability to clearly articulate the skill set that is unique to health services research is an unsatisfactory response to these talented students, and highlights the need to develop a common set of HSR competencies.

This manuscript describes an initial set of core competencies ("Version 1") for individuals trained at the doctoral level in HSR. The goal was to define the essential knowledge- and skills-based competencies expected of all HSR trainees, irrespective of areas of content specialization, method or disciplinary focus. Defining HSR doctoral competencies will (1) assist educators with developing and identifying the most important learning objectives for HSR training, (2) clarify for employers the skills and abilities of graduates of HSR training programs, and (3) give HSR trainees a unique professional identity.

### Defining "Competency"

Ideally, designing a training program begins with defining its competencies, delineating learning objectives specific to each competency, and then developing a structure for delivering educational experiences that fulfills the objectives [3]. The aim of this project was to focus on the first of these activities by defining core competencies that are common across doctoral programs in HSR. Doctoral level core competencies are defined as knowledge or skill-based assets that doctoral trainees in HSR should acquire during their training; they are the common outcomes across all training programs. Core competencies help to define professional role expectations and are likely to be broader in scope than individual program competencies.

Each competency has a set of learning objectives, which describe the approach for achieving the competency. Although the end-results (i.e., competencies) of training programs may be similar, the approaches used to attain them (i.e., learning objectives) may differ. This manuscript does not propose specific learning objectives, curricula, or methods that could be used for each competency.

#### Approach to Competency Development

We developed the core competencies by reviewing the literature and conducting a text analysis of accreditation self-studies submitted to the Council on Education for Public Health. Based on common themes identified in the self-studies, an initial draft of core competencies was developed and subsequently discussed at a conference of HSR educators. The Version 1 competencies presented in this manuscript are the result of this consensus conference. It should be emphasized that the methods used to generate these competencies were based on review of US training programs.

A search of the peer- and non-peer reviewed literature was conducted for all articles and reports that describe competencies or learning objectives in health services research (which for the purposes of this project also included health policy but not healthcare management) [4]. Literature from the education community [3,5] helped define the role competencies play in creating program learning objectives and curricula, and identified some of the problems associated with defining competencies. However, there was a dearth of literature on program competencies in health services research professions.

Because the focus of this project was on health services researchers, programs designed to train managers of health systems were considered outside the project scope [6-8], as were articles and reports that described competencies in the general public health workforce [9,10]. Excluding these groups, no literature was found that fully explicates the competencies of this field, even with the review of the MPH-level competencies for health policy and management developed by Association of Schools of Public Health [11].

The project team obtained accreditation self-studies of all schools of public health doctoral and master's level programs in health services research from the Council on Education for Public Health (CEPH) – a total of 27 schools and 55 programs. Masters programs were included in this review, because many programs did not distinguish in the accreditation self-study between doctoral and masters level training. Approximately 650 learning objectives were extracted from the self-studies, and then sorted into learning objective clusters using text analysis software that may be used to derive themes from text-heavy data [12]. The inter-observer reliability of the domain sort was tested by having two members of the research team independently assign learning objectives to clusters and then comparing assignments (inter-observer agreement was 93%). Disagreements were adjudicated by one investigator (CBF).

Based on this categorization of learning objectives, the project team drafted an initial set of core competencies that summarized the learning objective clusters. For example, scientific method and theory, literature review methods, and proposal development were three clusters that were ultimately associated with a single core competency: "pose innovative and important research questions, informed by systematic reviews of the literature, stakeholder needs, and relevant theoretical and conceptual models."

The draft version of the core competencies and their associated learning objective clusters were reviewed by the health services research faculties from the University of Washington and the Johns Hopkins Schools of Public Health. Their comments were used to produce a revised version. These materials were submitted to a geographically diverse batch of leaders of HSR training programs from across the United States in order to obtain the input of their faculty. These leaders and their programs were selected in consultation with staff responsible for HSR training programs funded by the Agency for Healthcare Research and Quality. The specific programs were selected to represent the major census divisions in the US, programs with and without CEPH-accreditation, and others that were or were not funded by the Agency for Healthcare Research and Quality.

The conference was held in the fall of 2005 and included 10 directors of HSR training programs, doctoral students, and representatives from AcademyHealth, AHRQ, CEPH, and public and private sector stakeholder organizations that are employers of HSR trainees. The group used a consensus conference process to revise the specific knowledge- and skills-based competencies that are common to all health services research professionals. A final version of competencies resulted from this consensus process, and is presented in this manuscript.

### The Core Competencies

The specific wording of each competency was intended to reflect the level of mastery and to identify the specific asset that HSR doctoral training should develop among its trainees. Table 1 provides specific wording for each of the 14 competencies, which form the core knowledge and methods of health services research. Learning objective content areas are also shown in the table in order to illustrate the HSR content relevant to attainment of the competency.

**Table 1:** Health Services Research Doctoral Level Core Competencies and Associated Learning Objective Content Areas

**#**	***Core Competency***	**Associated Learning Objective Content Areas**
1	Demonstrate ***breadth of HSR theoretical and conceptual knowledge ***by applying alternative models from a range of relevant disciplines.	Biomedicine Economics Epidemiology Informatics Management Sciences Political Science Psychology Sociology Statistics
2	Apply ***in-depth disciplinary knowledge and skills ***relevant to health services research.	Variable depending on the discipline or interdisciplinary area of specialization
3	Apply knowledge of the structures, performance, quality, policy, and environmental context of health and health care to ***formulate solutions for health policy problems***.	Access and Use Financing of Health Care Health Health Economics Health Policy Organization of Health Care Quality of Care
4	***Pose innovative and important health service research questions***, informed by systematic reviews of the literature, stakeholder needs, and relevant theoretical and conceptual models.	Scientific Method and Theory Literature Review Proposal Development
5	***Select appropriate interventional, observational, or qualitative study designs ***to address specific health services research questions.	Study Design for Interventions Observational Study Design Qualitative Research
6	Know how to ***collect primary health and health care data ***obtained by survey, qualitative, or mixed methods.	Survey Research Qualitative Research Primary Data Acquisition and Quality Control
7	Know how to ***assemble secondary data ***from existing public and private sources.	Health Informatics HSR Data Sources Secondary Data Acquisition and Quality Control
8	***Use conceptual models and operational measures ***to specify study constructs for a health services research question and develop variables that reliably and validly measure these constructs.	Measurement Theory and Methods Variable Construction
9	***Implement research protocols ***with standardized procedures that ensure reproducibility of the science.	Research Management
10	***Ensure the ethical and responsible conduct of research ***in the design, implementation, and dissemination of health services research.	Research Ethics
11	***Work collaboratively in multi-disciplinary teams.***	Teamwork
12	***Use appropriate analytical methods ***to clarify associations between variables and to delineate causal inferences.	Advanced HSR Analytic Methods Advanced Statistics Economic Evaluation Decision Sciences
13	***Effectively communicate the findings and implications of HSR ***through multiple modalities to technical and lay audiences.	Proposal Development Dissemination
14	***Understand the importance of collaborating with stakeholders***, such as policymakers, organizations, and communities to plan, conduct, and translate health services research into policy and practice.	Community Participatory Research Translating research into practice and policy

The list of 14 core competencies should be viewed in its entirety as a reflection of values and teaching goals of the HSR doctoral training programs, professional societies, and stakeholder organizations that contributed to this project. While some academic programs may emphasize specific core competencies more than others, it is the totality of the core competencies that makes the list unique to HSR. Individual core competencies are written in broad terms to allow individual variation and interpretation among programs. Some are expressed in such general language that they may be relevant to disciplines other than HSR. This is appropriate since some competencies (e.g. posing innovative research questions; ethical conduct of research) are central to conducting research in all fields, but this should not minimize the relevance of their inclusion in the core competencies for HSR.

### Implications and Next Steps

The competencies presented in this manuscript are a first step towards explicating the common knowledge and skill sets of health services researchers. They are presented to generate dialogue and debate both in the US and globally on the essential competencies for HSR. It is possible that non-US programs would view other competencies as core to the educational experience, such as comparative health systems research. Lack of inclusion of these international programs should be seen as a limitation of this work.

Further work is necessary to clearly identify the field's unique conceptual and methodological contributions to scientific inquiry. These efforts will undoubtedly help refine the HSR core competencies. Within the past two years, these competencies have been discussed and debated at a supplemental meeting to the 2007 Annual Research Meeting of AcademyHealth. At a 2008 conference sponsored by the Agency for Healthcare Research and Quality, approaches to how a particular program would deliver and evaluate them were examined by a group of HSR training program directors. They are published now to extend this dialogue to the global audience of health services research programs, employers of HSR trainees, and students.

Competencies can be an effective tool to guide the design of health services doctoral training programs and can contribute to the definition of the field of health services research. Stakeholder debates can elucidate HSR strengths and identify areas for further development – e.g. development of theories of organizational change to improve quality or better methods to take selection bias into account. To maximize the utility of core competencies, they should be embedded in experiential training as well as courses. Didactic course work is only a small part of doctoral education and much learning takes place during seminars, independent studies with faculty, research assistantships, and research field experiences. One of the next steps in thinking about curriculum development with the competencies will include suggestions for implementing experiential learning in doctoral training. This first foray into competency development should not indicate that HSR is ready for an accreditation process, as accreditation may have the unintended effect of stifling innovation in HSR training. However, this emerging framework could serve as a possible template for continuing education of HSR professionals as the specificity of HSR competence increases with further work.

The application of these competencies should not result in a set of "cookie cutter" training programs. In implementing the competencies, we expect that programs will consider the characteristics of their students, the expertise and interests of their faculty, and the opportunities presented by their research partners for field experiences. Programs should continue to offer training with emphasis in specific methods or topic areas. For example, one program may specialize in qualitative methods such as community-based participatory research, while another may concentrate on health economics.

The 14 core competencies provide an overview of the breadth of expertise that can be expected of all graduates of US-based HSR doctoral training programs and a minimum level of depth (i.e., mastery level). The specific level of competence in each educational domain, however, is a topic for each training program to grapple with. For example, we did not find unanimity that all HSR trainees should acquire an independent mastery level for primary and secondary data gathering studies. Instead, these competencies were worded to reflect an expectation of an intermediate level of competence – i.e., know how to apply or do something in a supervised setting. It would be expected that individual trainees would choose one of the two types of methodological approaches to gain independent expertise during their doctoral training.

The Version 1 core competencies presented in this paper are a product of work done in 2005 and should be considered a first draft. The competencies should be dynamic, changing as the field of health services research changes. For this reason it is likely that the core competencies will need to be revised on a periodic basis. We are publishing Version 1 of the HSR core competencies to generate discussion among a wider group of health services research professionals.

## Pre-publication history

The pre-publication history for this paper can be accessed here:


